# From aesthetics to ethics: Testing the link between an emotional experience of awe and the motive of quixoteism on (un)ethical behavior

**DOI:** 10.1007/s11031-022-09935-4

**Published:** 2022-03-21

**Authors:** Sergio Villar, Pilar Carrera, Luis Oceja

**Affiliations:** 1 Universidad Autónoma de Madrid (UAM), Iván Pavlov 6, 28049 Madrid, Spain; 2 Universidad Internacional de La Rioja (UNIR), Logroño, Spain

**Keywords:** Quixoteism, Awe, Prosocial behavior, Helping, Moral dilemma

## Abstract

According to the awe-quixoteism hypothesis, one experience of awe may lead to the engagement in challenging actions aimed at increasing the welfare of the world. However, what if the action involves damaging one individual? Across four experiments (N = 876), half participants were induced to feel either awe or a different (pleasant, activating, or neutral-control) emotion, and then decided whether achieving a prosocial goal (local vs. global). In the first three experiments this decision was assessed through a dilemma that involved to sacrifice one individual’s life, additionally in Experiments 2 and 3 we varied the quality of the action (ordinary vs. challenging). In Experiment 4, participants decided whether performing a real helping action. Overall, in line with the awe-quixoteism hypothesis, the results showed that previously inducing awe enhanced the willingness to sacrifice someone (Experiments 1, 2 and 3) or the acceptance to help (Experiment 4) when the decision involved engaging in challenges aimed at improving the welfare of the world.

Immanuel Kant (1724–1804) and Edmund Burke (1729–1797) investigated the concepts of the sublime and beauty and reached contrasting conclusions. On the one hand, Burke highlighted that the qualities of terror, strength, immensity, and obscurity make us feel insignificant in the face of magnificence and become less self-centered (Burke, 1757). On the other hand, Kant emphasized that the experience of the “dynamic sublime” leads us to appreciate our own power as humans and be able to transcend even the most terrifying aspects of our surroundings (Kant, 1790). In this regard, an emergent scientific study on this issue from the psychological perspective has addressed three issues. First, regarding the meaning and measure of the sublime experience, the current stream has virtually equated it to the emotion of awe and leaned toward Burke’s position (Keltner & Haidt, 2003; Shiota et al., 2007). Second, regarding the consequences of feeling this emotion, research has centered on the positive side. For example, the results suggest that awe promotes group coordination and cohesiveness (Shiota et al., 2007), generosity, willingness to volunteer to help other people, and providing actual aid to another individual (Piff et al., 2015; Rudd et al., 2012). Third, regarding the set of processes that may explain these positive outcomes, researchers have proposed the elicitation of supernatural beliefs (Van Cappellen & Saroglou, 2012), agency detection in random events (Valdesolo & Graham, 2014), self-diminishment (Piff et al., 2015; Bai et al., 2017), humility (Stellar et al., 2017a, 2017b), expansion of the perception of time (Rudd et al., 2012), reduced conviction in one’s opinions (Stancato & Keltner, 2019), and curiosity (Anderson et al., 2019).

With respect to these three issues, we claim in the present work that (a) awe also has a Kantian variant, (b) this awe experience may provoke negative outcomes, and (c) the motive of quixoteism is a psychological process that deserves attention in this line of research. Specifically, we predict that awe-eliciting stimuli may increase helping behavior and provoke morally dubious decisions such as the sacrifice of another individual’s life. We build our proposal upon the hypothesized link between an awe experience and the motive of quixoteism.

## The awe-quixoteism link

According to Batson’s Lewinian approach to the motives that may explain prosocial behavior, they diverge from their ultimate goal. That is, the ultimate goals of egoism, altruism, collectivism, and principlism are to increase one’s own welfare, to increase that of another specific individual, to increase that of a group and to uphold principles such as freedom or justice, respectively (Batson, 1994, 2011). Likewise, Oceja and collaborators proposed the existence of quixoteism, a motive with the ultimate goal of increasing the welfare of the world (Oceja & Salgado, 2008; Oceja & Salgado, 2013; Oceja & Stocks, 2017; Oceja et al., 2018, 2019; Salgado & Oceja, 2011; Villar et al., 2019, Villar, 2019). The concept world here does not necessarily refer to the planet Earth; rather, it refers to a transcendental and abstract idea that includes the sum of all that exists around us (Kant, 1781/1978). The social progress index might illustrate the kind of goals that involve the welfare of the world as a whole (Green, 2014).^1^

Additionally, Oceja and collaborators proposed that quixoteism is characterized not only by this ultimate goal but also by the instrumental goal of engaging in challenging actions (for the distinction between ultimate and instrumental goals, see Rokeach, 1973, and more recently, Oceja et al., 2019). For example, they found that when values associated with quixoteism are made salient by a situation or are central for people, the more challenging the action and the more global the goal, the greater the likelihood of pursuing a corresponding action.

They found this result with two different measures: the Transcendence-Change constellation and the Transcendental-Change profile. The first measure is formed by eight values taken from the Schwartz Values Survey (e.g., Schwartz, 2012): protecting the environment, exciting life, varied life, daring, social justice, curious, spiritual life and unity with nature (Villar et al., 2019, Studies 1 and 2). The second measure refers to the readiness to engage in challenges that can make the world a better place (Oceja et al., 2019; Studies 4 and 5).

Affective experiences can also be antecedents of social motives. For example, research on the empathy-altruism link supports that empathic concern toward an individual in need elicits an altruistic motivation that may lead to helping that individual (Batson, 1987, 2011; Batson et al., 2015). Furthermore, this *individualized* helping behavior may take place even at the expense of other individuals (Batson et al., 1995; Batson & Moran, 1999; Oceja & Jiménez, 2007; Oceja et al., 2014) and/or moral principles (Batson et al., 1995; Oceja, 2008). Our proposal resembles that line of research in two aspects. First, we test whether an awe-related emotional experience (i.e., feeling energetic, daring, amazed, curious, elevated, and ecstatic) elicits quixoteism, which may, in turn, lead to engaging in a challenging action to increase the welfare of the world. Second, this *transcendent* helping behavior led by the awe-quixoteism link may take place even at great expense to an individual (i.e., his or her sacrifice).

**Fig. 1:**
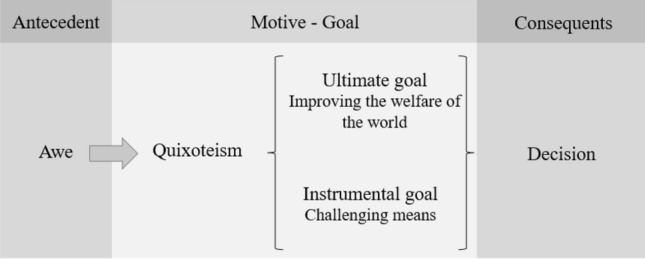
Explanatory diagram of the awe-quixoteism hypothesis

This particular experience of awe is related to Kant’s “dynamic sublime” and can be the emotional antecedent for quixoteism because its qualities of openness and transcendence relate to the instrumental and ultimate goals of quixoteism (i.e., engaging in challenging actions to improve the welfare of the world). Therefore, based on the conceptual analysis and the empirical findings referring to awe and quixoteism, we hypothesized their link and predicted that this kind of awe would increase the willingness to make a prosocial decision when that decision involves engaging in a challenge to increase the greater good (see Fig. 1). Moreover, we provide experimental evidence that might shed light on the processes underlying one possible paradoxical effect of awe: provoking an action that can be regarded as both prosocial and unethical.

## The present research

From the Lewinian approach, motivational forces are situational states that are not usually accessible to consciousness. We therefore cannot trust self-reports to reveal them; instead, we examine their role by manipulating their theoretical antecedents and the situational characteristics that are congruent with their nature (Batson, 2011). To test our predictions, we need to induce awe while contrasting with the induction of other emotional experiences, and present participants a controlled situation with a decision that varies in the characteristics associated with quixoteism (challenging and global action).

With respect to the induction of awe, two of the most common elicitors are nature (e.g., Cohen et al., 2010; Joye & Bolderdijk, 2015) and art (e.g., Gabrielsson, 2011; Pilgrim et al., 2017). Music has also been used to induce emotion (e.g., Cowen et al., 2020; Silvia et al., 2015). Our research, using music as an elicitor, prevents confusing effects that are especially relevant for the awe-quixoteism link; that is, images of nature or art may inadvertently activate concepts related to the instrumental and ultimate goals of quixoteism. Therefore, we conducted a separate pilot study to ensure that the three music clips used in this paper induced the desired emotions.

### Pilot study

One hundred thirty-eight participants (46 per condition) listened to one of three pieces of audio: one awe-eliciting (*Wild Side* by Roberto Cacciapaglia), one pleasant (*Gymnopédie No. 1* by Erik Satie), and one activating (*Toccata Op. 11*, by Sergueï Prokofiev). They then used a 7-point scale (1 = not at all, 7 = extremely) to indicate the extent to which they felt amazed, ecstatic, elevated, energetic, daring, curious, and pleasant. As shown in Table 1, the awe-eliciting audio clip produced greater levels of amazed, ecstatic, and elevated feelings than the pleasant and activating audio clips and greater levels of daring, curious, and energetic feelings than the pleasant audio clip. Therefore, the results supported our characterization of the audio clip as awe-eliciting.

**Table 1:** Mean scores (SDs) for self-reported emotional states in a previous pilot study (7-point scale)

	Awe-eliciting audio (N = 46)	Pleasant Audio (N = 46)	Activating audio (N = 46)
Elevated	5.33_a_ (1.58)	3.33_b_ (1.79)	3.35_b_ (1.79)
Ecstatic	4.37_a_ (1.90)	3.09_b_ (1.74)	3.22_b_ (1.78)
Amazed	4.43_a_ (1.62)	3.43_b_ (1.71)	3.65_b_ (1.58)
Daring	4.39_a_ (1.71)	2.67_b_ (1.38)	4.26_a_ (1.90)
Curious	4.48_a_ (1.60)	3.67_b_ (1.81)	4.17_a_ (1.89)
Energetic	4.89_a_ (1.57)	3.28_b_ (1.64)	5.20_a_ (1.60)
Pleasant	6.37_a_ (0.97)	6.20_a_ (1.19)	4.17_b_ (1.69)

Means in the same row that do not share the same subscript differ at p < 0.05 in a between-subject t-test

Then, in four studies, we asked the participants to listen to the awe-eliciting music vs. pleasant music (Studies 2 and 4), activating music (Study 3), or no music (Studies 1, 2 and 3). In the first three studies, we presented the participants with a moral dilemma that involved killing one individual to obtain a local vs. global goal (Studies 1 and 3) through low vs. high challenging action (Studies 2 and 3). In Study 4, we presented the participants with the opportunity to perform an actual helping behavior oriented to achieve a local vs. global goal.

These studies allowed us to test whether the induction of an experience of awe characterized by feelings of openness and transcendence led the participants to make prosocial decisions that were in line with the instrumental and ultimate goals of quixoteism: engaging in challenges that may increase the welfare of the world (Fig. 1). In addition, in the first three studies, we tested whether this relation holds even when such a decision involves an unethical behavior such as sacrificing someone. In the fourth study, we additionally tested whether the induction of this awe reduced helping when it was not in line with the motive of quixoteism.

The a priori power analysis (G*Power 3.1; Faul et al., 2009) suggested a minimum sample size of 128 or 158 participants to detect a medium size effect (*f* = 0.25), *α* = 0.05, 1-*β* = 0.80 for fixed effects ANOVAs with factors that include two, four, six or twelve conditions. The four studies of this work fulfilled those requirements. In our studies, the sample sizes were consistent with those of previous studies on quixoteism (e.g., Oceja et al., 2018, 2019; Villar et al., 2019) and awe experience (e.g., Bai et al., 2017; Gordon et al., 2017; Stellar et al., 2017a, 2017b). These programs of research consistently obtained significant effects with similar sample sizes to ours, allowing us to expect a medium size effect. All the data that support this work are accessible in the Open Science Framework (https://osf.io/8ad5f/?view_only=25caf5049b4e4272b57a5227f7e2fae5).

## Study 1. The awe-quixoteism link: Testing the relevance of the ultimate goal

**Fig. 2:**
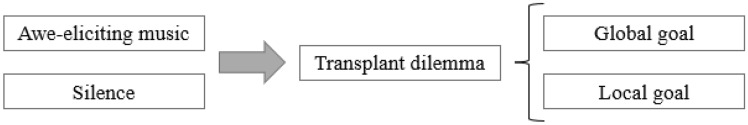
Experimental design of Study 1

In Study 1, we examined whether inducing awe led to a focus on the goal of improving the welfare of the world, even at the expense of an individual’s life. Specifically, we asked the participants to read a moral dilemma based on the Transplant Dilemma (Foot, 1967), where they had to decide whether to sacrifice a person for the greater good. Half of them read the case while listening to the awe-eliciting music, whereas the other half read it in silence. The dilemma always involved the decision of sacrificing a person for the greater good; for half of the participants, that greater good involved a more local goal (i.e., save many people), whereas for the other half, it involved a more global goal (i.e., to save the world). We intentionally discarded the possibility of committing a self-sacrifice to exclude mechanisms related to the self, such as being regarded as a heroic or virtuous person. Therefore, the participants were randomly assigned to one of the subsequent four conditions: 2 (affective experience: awe vs. silence) × 2 (ultimate goal: local vs. global) between-subject factorial design (Fig. 2).

### Hypothesis

In line with the awe-quixoteism hypothesis, we expected the awe-eliciting music to enhance the proneness to sacrifice when the ultimate goal was global. Namely, we predicted a 1 vs. 3 pattern (planned comparisons): awe-eliciting music & global goal vs. the other three conditions. It is noteworthy that this pattern did not equal the interaction between the variables. Indeed, the significant interaction was not sufficient because the crossed effects might not support the awe-quixoteism hypothesis, and it was not necessary because the 1 vs. 3 pattern could be found without interaction.

## Method

### Participants and procedure

Two hundred sixteen students (54 per condition; 181 female, *M*_age_ = 19.35, *SD* = 1.95) completed the experiment using computers placed in cubicles with headphones. We used Qualtrics software.

The participants gave consent and reported their demographics. Next, the participants in the awe-eliciting condition were asked to read a case scenario (a dilemma) while listening to awe-eliciting music (*Wild Side* by Roberto Cacciapaglia), whereas the participants in the control condition were asked to read only a case scenario. As mentioned above, we previously tested the stimuli in an independent pilot study.^2^

The participants read one of two versions of the Transplant Dilemma (Foot, 1967). They were asked to imagine being a doctor who must decide whether to sacrifice the life of a patient by surgically removing a gland that secretes a unique hormone capable of healing a new mortal flu strain that is devastating the world (one version) vs. the lives of many men and women (the other version).^3^

Immediately after the dilemma, the screen first presented two options: “*No, I would not perform the surgery*” and “*Yes, I would perform the surgery with a certain probability of success*”. The procedure was as follows. If the participants decided not to sacrifice the patient, the application moved to the next step. If they decided to sacrifice the patient, they were asked first whether they would make the sacrifice with a 0.1 probability of success; if their answer was “Yes”, the application moved to the next step. If the answer was “No”, the same question but with a 0.3 probability of success was presented. The same process occurred for probabilities of 0.5, 0.7, 0.8, 0.9 and finally 100%, with each question on a separate page.

**Fig. 3:**
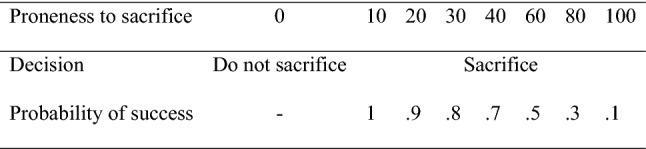
Coding of the ‘proneness to sacrifice’ variable. A lower required probability of success means a higher proneness to sacrifice

Once they answered that they would sacrifice the patient with a certain probability, the application moved to the next step and the music ceased. We elaborate this process by addressing three issues. First, giving all the probabilities of success at once would probably lead the participants to choose the safest option (100% success). Second, they did not know whether the procedure would offer a new probability or end. Third, they could not anticipate the value of the next probability. We recoded the scores in a proneness to sacrifice variable as the dependent variable by subtracting the offered probability from 110 to obtain an 8-point scale that ranges from 0 (no sacrifice) to 100 (sacrifice with the minimum probability, 0.1). Figure 3 presents the correspondence between the options and the scores of the 8-point scale.

## Results

Sixty-two percent of the sample decided to perform the surgery. Specifically, 23.1%, 31% and 7.9% sacrificed when the success probability was lower than or equal to 0.3, between 0.5 and 0.7, and equal to or higher than 0.8, respectively.

**Fig. 4:**
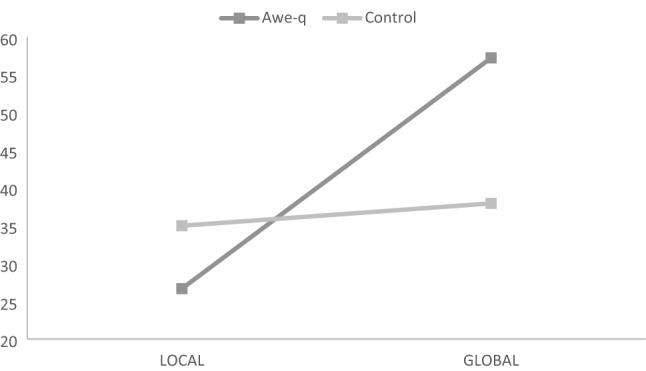
Interaction between the affective experience (awe vs. control) and the ultimate goal (local vs. global) in Study 1

We tested the influence of awe-eliciting music and the ultimate goal on the proneness to sacrifice. A two-way ANOVA showed a significant effect of the ultimate goal variable, *F*(1,214) = 11.76, *p* = 0.001, *η*_*p*_^*2*^ = 0.053, and a significant interaction between both variables, *F*(1,214) = 7.97, *p* = 0.005, *η*_*p*_^*2*^ = 0.036. As shown in Fig. 4, the participants who were listening to the awe-eliciting music reported higher proneness to perform the surgery when the goal was global (*M* = 57.22, *SD* = 38.48) than when it was local (*M* = 26.67, *SD* = 34.75); *t*(106) = 4.33, *p* < 0.001, *d* = *0.*61. This effect was not found among the participants who completed the dilemma in silence: *Ms* = 35.00 and 37.96, *SDs* = 35.65 and 34.66, for local and global, respectively; *t*(106) = 0.44, *p* = 0.66, *d* = *0.*09.

Finally, we conducted a 1 (awe-eliciting & global goal) vs. 3 (the other three conditions taken as a whole) planned comparison analysis. In line with the awe-quixoteism hypothesis, the difference was significant, *t*(212) = 4.25, *p* < 0.001. Further analyses showed that this 1 vs. 3 pattern was not significant when using any of the other three conditions as the target, *t*_*s*_(212) < 1.00, *p*_*s*_ > 0.30.

## Discussion

Participants of Study 1 faced the dilemma of attaining a prosocial goal through the acceptance of an action that involved the moral cost of sacrificing one individual. The results showed that only the combination of (a) provoking the emotional experience of awe and (b) depicting the final goal as saving the world increased the acceptance of the morally dubious action. This increase was not found when the experience of awe was not provoked and/or the goal was to save many people. We reason that this outcome supports the link between the emotion of awe and quixoteism, a motive with the ultimate goal of increasing the welfare of the world. Observing a similar emotion-motive link, Batson and collaborators (1995, 1999) found that eliciting the emotion of empathic concern can lead people to act unfairly only when the outcome is aligned with the ultimate goal of altruism: to enhance the welfare of the individual for whom empathic concern is felt.

Our proposed awe-quixoteism link involves one step further: the likelihood of making a morally dubious decision can increase when eliciting awe is combined with aligning the instrumental (engaging in a challenging action) and ultimate goals (saving the world) of quixoteism. Studies 2 and 3 address this issue.

## Study 2. The awe-quixoteism link: Exploring the relevance of the instrumental goal

**Fig. 5:**
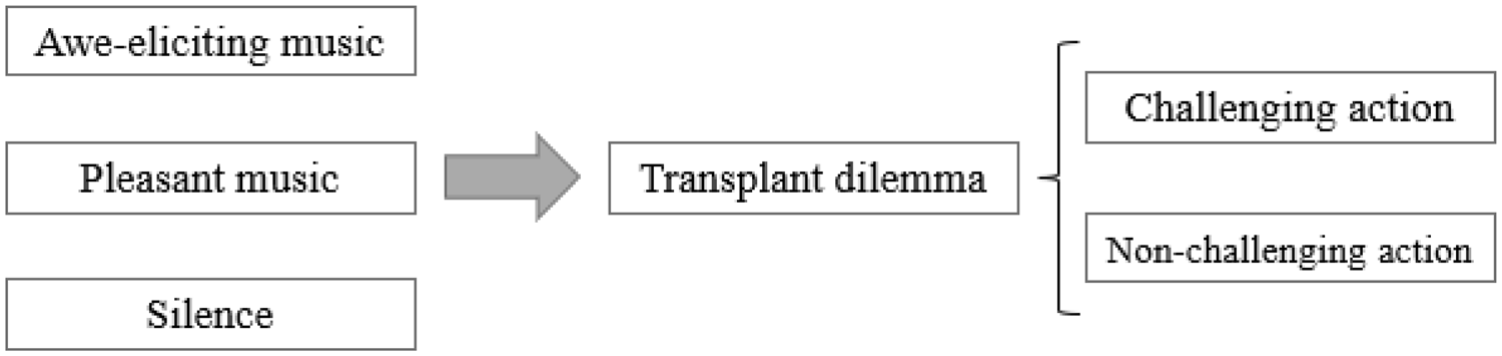
Experimental design of Study 2

In Study 2, we added a new control condition where the participants listened to pleasant but not awe-eliciting music. This allowed us to test whether the results found in Study 1 were due solely to the positive quality of the feelings induced by the awe-eliciting music. Furthermore, regarding the awe-quixoteism hypothesis, we included the instrumental goal (i.e., embarking on a challenging action) as an independent variable. On this occasion, to perform the operation required to take a trip either to an unknown and distant place (a challenging action aligned with the instrumental goal of quixoteism) or to a known and nearby place (a nonchallenging action). This time, we kept constant the final global goal of saving the world. As shown in Fig. 5, the participants were randomly assigned to one of the six between-participants conditions within a 3 (affective experience: awe vs. pleasant vs. silence-control) × 2 (instrumental goal: challenging vs. nonchallenging action) factorial design.

**Fig. 6:**
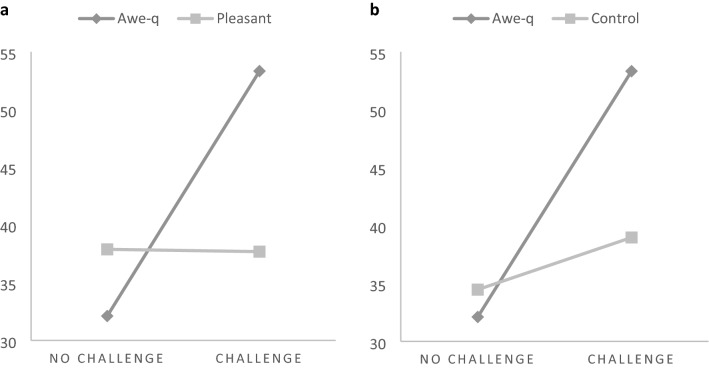
**a** and **b** Interaction between the affective experience (awe vs. pleasant) (awe vs. no music) and the instrumental goal (no challenge vs. challenge). Study 2

### Hypothesis

We expected the highest proneness to perform the surgery in the condition that included the experience of awe and the challenging action, that is, a 1 vs. 5 pattern (planned comparisons). Therefore, we did not expect this effect when there was no background music or the music was *just* pleasant or when the trip was specifically depicted as nonchallenging.

## Method

### Participants and procedure

Two hundred forty-five students (204 female, *M*_age_ = 19.29, *SD* = 2.40) followed an experimental procedure very similar to that of Study 1. Fourteen participants were excluded because of missing data, which left 231 participants in the final sample (38–39 per condition, 189 female, *M*_age_ = 19.28, *SD* = 2.47). With respect to the affective experience, the participants listened to either pretested awe-eliciting music (*Wild Side*, see Study 1) or pleasant music (*Gymnopédie No. 1* by Erik Satie) or did not listen to any music while completing the dilemma. In the previous pilot study, the pleasant music produced a high level of pleasantness but low levels of emotions related to awe (see Table 1).

The program displayed the dilemma used in Study 1 with one variation: the difference between the two versions referred to the instrumental goal instead of to the ultimate goal. In the challenging condition, the participants had to “*take a trip to a distant and unknown place*” to perform the surgery, whereas in the nonchallenging condition, they had to “*take a trip to a nearby and known place*”. All the participants were told that the ultimate goal of performing the surgery was to save the world. Next, the participants decided whether to sacrifice the patient following the procedure used in Study 1. In the control check completed at the end of the experiment, the participants answered “*How thrilling do you think the trip required to perform the surgery is*?” on a scale ranging from 1 (not thrilling at all) to 7 (extremely thrilling).

## Results

A total of 71.9% of the whole sample decided to perform the surgery: 19.1%, 35.9% and 16.9% sacrificed the patient when the success probability was 0.3 or lower, between 0.5 and a 0.7, and greater than or equal to 0.8, respectively. In line with the manipulation of the instrumental goal, the results showed that traveling to an unknown and distant place was considered more thrilling, *M* = 4.50, *SD* = 1.75, than traveling to a known and nearby place, *M* = 3.65, *SD* = 1.86; *t*(229) = 3.60, *p* < 0.001, *d* = *0.*95.

The average values of the proneness to sacrifice variable within each condition are shown in Table 2. As in Study 1, this variable ranges from 0 (participants who did not perform the surgery) to 100 (participants who sacrificed the patient with 0.1 probability of success). Regarding the effect of the instrumental goal, a 2 × 3 ANOVA revealed a marginally significant effect: the participants who read about the need to embark on a challenging trip to perform the surgery reported a higher proneness (*M* = 43.33, *SD* = 33.50) than those who read about the nonchallenging trip (*M* = 34.82, *SD* = 33.92); *F*(1,230) = 3.71, *p* = 0.055, *η*_*p*_^*2*^ = 0.16 (see Fig. 6a and b). Neither the effect of the interaction nor the main effect of the affective experience were significant.

**Table 2:** Means (SDs) for conditions in Study 2

	Challenge	No challenge
Awe	53.33_a_ (32.15)	32.11_b_ (29.52)
Pleasant	37.69_b_ (34.60)	37.89_b_ (37.21)
Silence	38.97_b_ (32.27)	34.47_b_ (35.24)

Means with different subindexes differed at p ≤ .05

In line with the awe-quixoteism hypothesis, we predicted that the combination of awe-eliciting music and a challenging trip would evoke the highest proneness to sacrifice. We tested this by comparing the target condition (awe-eliciting music & challenging trip) with the other five conditions (1 vs. 5 planned comparison analysis). As shown in Table 2, the participants in this condition were more prone to perform the surgery than the participants in the other five conditions taken as a whole, *t*(225) = 2.90; *p* = 0.004. This difference was not found when the target condition was any of the other five conditions; *ts*(225) < 1.41; *ps* > 0.16.

## Discussion

In Study 2, we manipulated the instrumental goal to test whether the more challenging the means are, the more likely a person is to perform a surgery that involves sacrificing one person to increase the welfare of the world. Importantly, based on the awe-quixoteism link, we predicted that this would be the case only when there was awe-eliciting background music.

The results of Study 2 ruled out that the higher likelihood of performing the action was due to the reactions provoked by eliciting a positive affective experience or depicting the means and the final objective as challenging and prosocial, respectively. First, presenting background music that evoked a pleasant affective experience did not increase the likelihood of accepting the sacrifice, regardless how the means and the final objective were depicted. Second, awe-eliciting music did not increase this likelihood when the means was explicitly depicted as not challenging. Therefore, in line with the awe-quixoteism link, the enhancing effect was found only when awe was elicited and the depiction of the means and final objective were aligned with the instrumental (challenging) and final (saving the world) goals of quixoteism, respectively (Oceja et al., 2019). In Study 3, we combined the manipulation of the ultimate and instrumental goals in the same design.

## Study 3. The awe-quixoteism link: Exploring the combination of the ultimate and instrumental goals

**Fig. 7:**
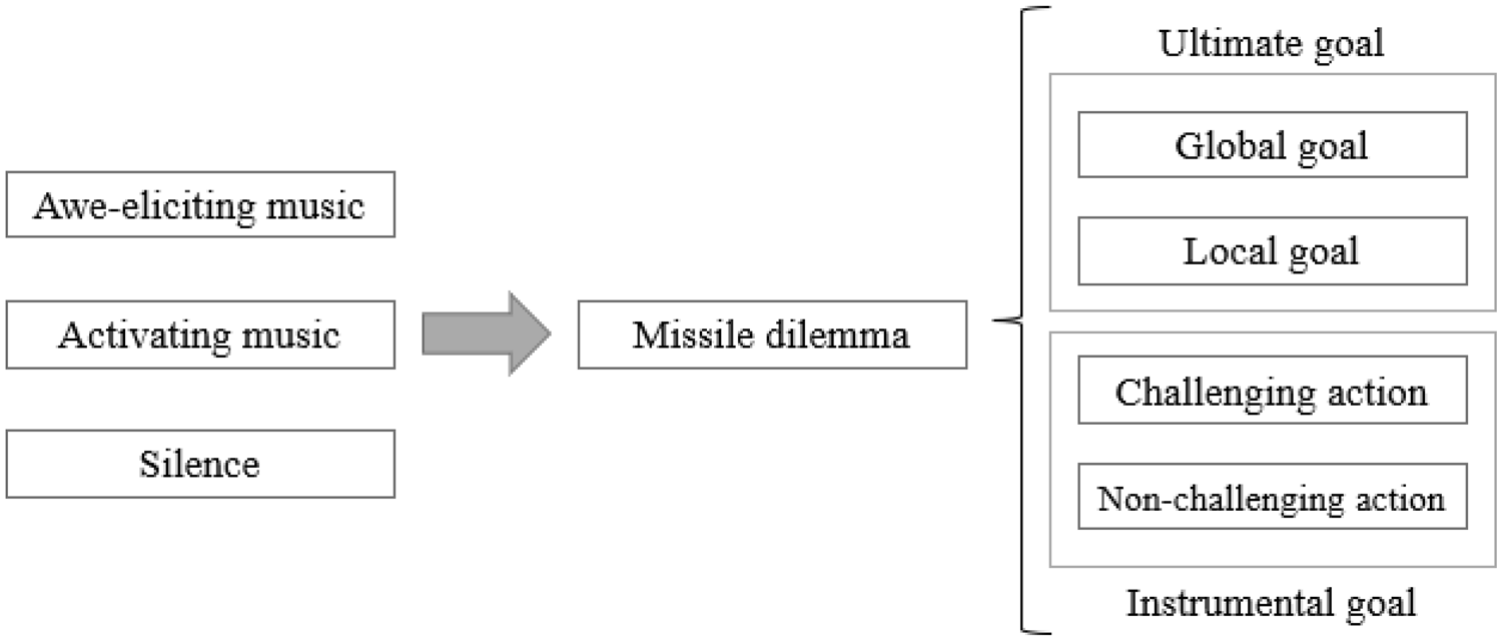
Experimental design of Study 3

In Study 3, we maintained the same structure used in Studies 1 and 2 while including both the ultimate and instrumental goals as independent variables. In addition, instead of using pleasant music as a control, we now included activating music to assess whether the effects found could be due to the arousal associated with our awe-eliciting music. Therefore, we conducted a 3 (affective experience: awe-eliciting vs. activating vs. silence) × 2 (ultimate goal: global vs. local) × 2 (instrumental goal: challenging action vs. nonchallenging) between-subjects design (see Fig. 7).

### Hypothesis

We expected that the target condition (awe-eliciting music, improving the welfare of the world as the ultimate goal, and engaging challenges as the instrumental goal) would result in the highest score in the proneness to sacrifice variable; that is, a 1 vs. 11 pattern.

## Method

### Participants

Two hundred sixty-seven students (230 female) completed the experiment. Fifteen participants were excluded because technical difficulty did not allow them to listen to the music properly, obtaining a final sample of 252 participants (21 per condition, 215 female, *M*_*age*_ = 19.60, *SD*_*age*_ = 1.68).

### Materials and procedure

After reporting their demographics, the participants listened to the music. The awe-eliciting clip was the same as that used in Studies 1 and 2 (*Wild Side*, by Roberto Cacciapaglia). The activating music (*Toccata Op. 11*, by Sergueï Prokofiev) was also tested in the pilot study, obtaining the lowest scores for pleasantness and high scores in activation-related terms (see Table 1). A third of the participants read the dilemma in silence.

We used a dilemma with the same structure as the one used in Studies 1 and 2, although with some variations. Inspired by the torpedo dilemma (see Waldmann & Dieterich, 2007), the participants were asked to imagine that they were a senior official who should make an important decision. Namely, an atomic missile is out of control and it will crash into a plane with 10 passengers (local goal) or into the Earth’s surface (global goal), depending on the ultimate goal condition. The participants were then presented the opportunity to travel to the army headquarters to take remote control of a light aircraft, crash it into the missile and, consequently, divert the missile to outer space. Regarding the instrumental goal, the army headquarters was located in a known and nearby place (nonchallenge condition) or in an unknown and distant place (challenge condition). Unfortunately, making the decision to crash the light aircraft into the missile will kill the aviator. With respect to the dependent variable (proneness to sacrifice), following exactly the same procedure used in Studies 1 and 2, those participants who decided to crash it were progressively asked about the probability of the decision being successful.

## Results

A total of 83.7% of the final sample (211 participants) decided to sacrifice the aviator: 36.1%, 37.3%, and 10.40% sacrificed the aviator when the success probability of diverting the missile was 0.3 or lower, between 0.5 and 0.7, and greater than or equal to 0.8, respectively.

An ANOVA for the proneness to sacrifice variable including the three independent variables (affective experience, instrumental goal, and ultimate goal) revealed a significant effect of the ultimate goal, *F*(1,251) = 15.74, *p* < 0.001, *η*_*p*_^*2*^ = 0.062. Specifically, the reported proneness to sacrifice the aviator was higher among those participants in the global-goal conditions (*M* = 64.60, *SD* = 34.79 on a scale ranging from 0 to 100) than among those in the local-goal conditions (*M* = 47.38, *SD* = 34.46). Furthermore, although the interaction between the instrumental goal and the affective experience did not reach significance [*F*(2,251) = 2.53, *p* = 0.082, *η*_*p*_^*2*^ = 0.021], the results showed that in the awe conditions, the more challenging the means were (i.e., having to travel to a distant and an unknown place vs. a close and known place), the higher the proneness to sacrifice the aviator: *Ms* = 68.57 vs. 52.14, *SDs* = 34.26 and 35.85, respectively), *t*(82) = 2.15, *p* = 0.035.

Finally, regarding the awe-quixoteism hypothesis, as shown in Table 3, the participants in the target condition (awe-eliciting music, challenging travel, and global goal) showed the highest proneness to sacrifice the aviator (*M* = 81.90). To specifically test our hypothesis, we conducted all the possible 1 vs. 11 planned comparisons, varying the target condition. As predicted, only the one for the “awe-challenge-global” as the target condition reached significance, *t*(240) = 3.60, *p* < 0.001, whereas the other 10 contrasts did not, *t*s(240) < 1.64, *ps* > 0.10.

**Table 3:** Means (SDs) for conditions in Study 3

	Global		Local	
	Challenge	No challenge	Challenge	No challenge
Awe	81.90 (28.92)	56.19 (35.56)	55.24 (34.59)	48.10 (36.55)
Activation	67.14 (30.36)	54.29 (39.70)	50.48 (34.85)	44.76 (33.85)
Silence	64.29 (35.58)	63.81 (34.57)	35.71 (37.76)	50.00 (29.50)

## Discussion

The results of Study 3 showed that presenting awe-eliciting music did not increase the likelihood of sacrificing the aviator when the means and the final objective were depicted as non-challenging and local, respectively. Furthermore, presenting only activating music or not presenting music did not increase the likelihood of the “prosocial sacrifice” when the means and final objective were depicted as challenging and global, respectively. Therefore, extending Studies 1 and 2, Study 3 showed that the elicitation of the awe emotional experience enhanced the likelihood of the “prosocial sacrifice” only when the depiction of the means and final objective were aligned with the instrumental (challenging) and ultimate (global) goals of quixoteism (Oceja et al., 2019; Villar et al., 2019).

## Study 4. The impact of the awe-quixoteism link on actual behavior

In our fourth experiment, we removed the dubious moral side effects of damaging one individual and centered on testing the influence of the awe-quixoteism link on actual prosocial behavior (i.e., committing to volunteer on an existing charity action). Therefore, we conducted a 2 (affective experience: pleasant vs. awe) × 2 (final goal: global vs. local) between-subjects experiment in which the participants were asked to volunteer in an actual prosocial initiative (i.e., a yearly organized food bank).

### Hypothesis

 We expected that previous exposure to awe-eliciting music would increase actual volunteering, mainly when the initiative was framed in line with the ultimate goal of quixoteism (i.e., improving the welfare of the world).

## Method

### Participants and procedure

One hundred forty-eight students (108 female, *M*_age_ = 19.09; *SD* = 4.06) individually completed the experiment in isolated cubicles (37 participants per condition). When entering the cubicle, the experimenter explained that the instructions to follow the procedure were in a PowerPoint presentation with five slides that they could view at their own pace. The first slide explained that the study was conducted in collaboration with a set of organizations that had elaborated various kinds of audiovisual materials and wanted to test how they would be perceived by the potential audience (students). On the second slide, the participants read that they were randomly assigned to listen to an audio clip designed to be presented as background music in an advertisement. They were then asked to sign the consent statement and put it in an envelope if they agreed to proceed (all participants did). The third slide presented one link along with the instruction “Please put the headphones on, click the link and listen to the music”. Depending on the condition, this link included either the pleasant music or the awe-eliciting music used in previous studies.

When the music finished, the participant read that the organization that had designed the audio clip had also written a letter to thank them for their collaboration and briefly described its activities. In this letter, all the participants read that the organization was an NGO that holds an annual campaign to collect nonperishable food. Indeed, the participants read that the next campaign would be the second weekend of the next month, and they were offered the opportunity to collaborate by spending some time in one of the places located to collect the food (from 1 to 4 h). It was made clear that the collaboration was voluntary.

Those participants who wanted to collaborate completed the corresponding lines indicating the number of hours (on a scale ranging from 1 to 4 h) and some personal contact information, such as name, age, and email. Those who did not want to collaborate just had to leave these lines blank. The letter was identical in each condition with one exception: in one case, the participants read “We are the World Food Bank and our objective is to eradicate hunger from the world” (global goal); in the other case, they read “We are the [City Name] Food Bank and our objective is to provide food to those families in [City Name] that need it” (local goal). A final slide asked them to open the door of the cubicle and call the experimenter, who approached and debriefed them.

## Results

**Fig. 8:**
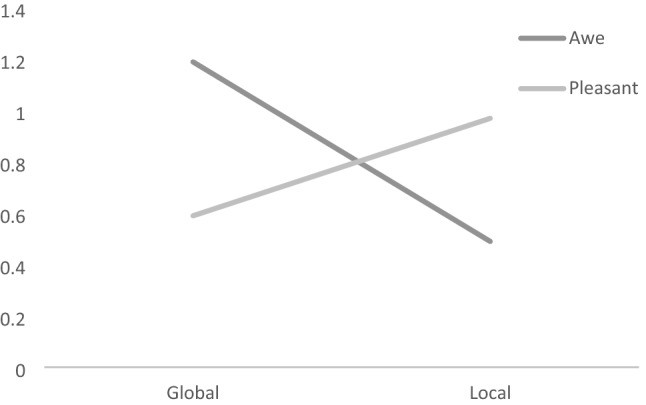
Interaction between the affective experience (awe vs. pleasant) and the instrumental goal (global vs. local). Study 4

Fifty-seven participants (38.5%) agreed to collaborate with the NGO, and the average donation time was almost one hour (*M* = 0.81, *SD* = 1.16). A two-way ANOVA with the induced emotional experience (awe vs. pleasant) and the description of the ultimate goal (world vs. families) as independent variables and the donated number of hours (ranging from 0 to 4) as the dependent variable revealed a significant interaction between the emotional experience and the description of the goal, *F*(1,147) = 8.33, *p* = 0.004, *η*_*p*_^*2*^ = 0.055 (see Fig. 8). This interaction shows that the participants who previously felt awe donated significantly more time when the ultimate goal was global (i.e., to eradicate the hunger in the world) (*M* = 1.19, *SD* = 1.35) than when it was local (i.e., to feed deprived families from [City Name]) (*M* = 0.49, *SD* = 0.84); *t*(72) = 2.69, *p* = 0.009. In contrast, for the participants who previously felt a pleasant emotional experience, the pattern was the opposite: donating more time when the ultimate goal was local (*M*s = 0.97 vs. 0.59, *SD* = 1.19 and 1.12), although the difference did not reach significance; *t*(72) = 1.41, *p* = 0.163.

## Discussion

Connecting the research on the influence of awe (Piff et al., 2015; Rudd et al., 2012) and quixoteism (Oceja et al., 2018; Villar et al., 2019) to actual prosocial action, Study 4 highlighted the relevance of the type of goal associated with the action. The positive effect of eliciting awe was higher when this goal was explicitly depicted as improving the welfare of the world (global). Instead, when the goal was to improve the welfare of a specific group (local), the effect of awe on prosocial action did not differ from that provoked by a pleasant emotional experience. In line with the results obtained in the previous three studies, these results support the awe-quixoteism hypothesis.

## General discussion

The psychosocial approach has typically portrayed the set of self-transcendent emotions as important because they can reduce the orientation toward the self and, consequently, promote action that involves positive outcomes for others (Stellar et al., 2017a, 2017b). These emotions include the experiences of compassion (Batson, 2011), gratitude and appreciation (McCullough et al., 2001), elevation, inspiration, and admiration (Keltner & Haidt, 2003), love (Fredrickson, 2013; Shaver et al., 1996), and kama muta (Fiske et al., 2019). Within this set, the experience of awe is an essential exemplar that can enhance the welfare of others (e.g., Keltner & Haidt, 2003; Shiota et al., 2007). In this work, we concur with this point of view, although we centered on the connection of a particular case of awe that is overall characterized by feeling energetic, daring, amazed, curious, elevated, and ecstatic with the motive of quixoteism. This approach led us to address two questions. Can this awe increase the willingness to make a decision that involves improving the welfare of the world at the expense of sacrificing a person? Does the positive effect of this awe on actual prosocial behavior depend on the extent to which it is oriented to improve the welfare of the world?

Taken together, the results of the first three studies suggested a positive answer to the first question. Furthermore, in line with our awe-quixoteism hypothesis, awe did not increase the proneness to sacrifice one individual when the means were depicted as not challenging and/or the goal was not related to the world. To address this first question, we had to turn to the use of moral dilemmas. This technique has been widely used when examining the factors that influence decisions that involve the traditional moral dilemma of whether harming a person is justified to save several others (see, for example, the research on the classic trolley problem; Thomson, 1985; Cushman & Greene, 2011). However, critics have reasonably objected that these hypothetical situations may promote decisions aimed at adjusting to the normative appropriate rules set in the experimental context (Lerner, 2003). This criticism prevents us from inferring that the response to the moral dilemma allows us to predict future *real* individuals’ behaviors (Aguilar et al., 2013). That is not actually our purpose. Instead, led by the awe-quixoteism hypothesis, we aimed to disentangle whether there is a specific combination of factors that tip the balance when solving the dilemma.

Regarding the second question, the results of a fourth study showed that the induction of awe increased the actual decision to perform helping behavior only when it was framed as a means of increasing the welfare of the world. Indeed, when helping was framed as a means of increasing the welfare of a local group in need, the induction of awe was less effective than inducing a pleasant emotional experience. These results are coherent with previous research that shows that either the salience or the centrality of a set of values linked to quixoteism increase the support for actions focused on making the world a better place (Oceja et al., 2018, 2019; Salgado & Oceja, 2011).

## Implications, limitations and future directions

Why did awe provoke this outcome? We proposed and tested that this effect was due to the link of a particular experience of awe with quixoteism, a motive characterized by the combination of two goals: engaging in challenges (instrumental goal) that may increase the welfare of the world (ultimate goal) (Oceja, et al., 2019; Villar et al., 2019). Note that we deliberately referred to a particular awe emotional experience. This experience—comprised of feeling energetic, daring, amazed, elevated, curious, and ecstatic—is in line with Kant’s “dynamic sublime”, being different from the experience of awe based on Burke’s view (Keltner & Haidt, 2003; Shiota et al., 2007). However, these two types of awe are closely related to each other, and both are associated with aesthetic appreciation, following the semantic space proposed by Cowen and Keltner (2017).

Aligned with a psychological constructionist perspective on emotions (Russell, 2009), the conceptual framework of Cowen and Keltner (2017) highlights the gradients of and variations between emotional experiences. This allows us to understand that the subtypes of awe may have different mechanisms. Indeed, in their proposal and study of self-diminishment, Piff et al., (2015, p. 896) stated “whereas we focused on the mediating role of the small self, other plausible mechanisms linked to awe should be tested”. We agree with these authors and propose the motive of quixoteism as another mechanism connected to some facets of the complex experience of awe. This opens venues for future research. Namely, feeling smaller and insignificant seems to be incompatible with the instrumental goal of quixoteism (i.e., embarking in a challenge); however, self-diminishment and quixoteism draw attention to something greater (i.e., the world as a whole) and away from personal day-to-day concerns. This suggests that the sense of self-diminishment and the motive of quixoteism could combine to boost action oriented at achieving a greater good. Comparing kinds of awe and examining (a) their mutual relationships and (b) the psychological processes that they may provoke deserves a future program of research.

We designed the first three studies aimed at highlighting the dilemma quality of the decision; for ethical reasons, we could not obtain behavioral responses. In contrast, we designed the fourth study to highlight the behavioral quality of the decision. In line with the awe-quixoteism link, the results in Study 4 showed that awe-eliciting music enhanced behavioral commitment only when the goal was explicitly depicted as increasing the welfare of the world (vs. the welfare of a specific deprived collective). Regarding this effect on prosocial behavior, the participants committed to volunteer by signing up and providing us with their personal contact information. We do not know to what extent they finally showed up. Indeed, this issue regarding the duration of the effect of a specific emotion-motive link on actual long-term behavior deserves more attention in future psychological research.

The finding that the effect of awe on the willingness to sacrifice someone is strongest when the end is explicitly depicted as global and the means as challenging (i.e., the awe-quixoteism hypothesis) calls for more attention to be given to the important theoretical difference in instrumental and ultimate goals. This is in line with those who suggest that forming meaningful combinations of values enlarges their explanatory power (Lönnqvist et al., 2006); we understand those means-ends relationships that match specific emotion-motive links to be meaningful. One limitation of our work is that we did not directly address the distinction between global and local ends. Although we used terms that should provoke this distinction and the overall results were in line with the awe-quixoteism hypothesis, future research is needed to define those parameters in which global and local ends differentiate from each other. More research is also necessary on the specific characteristics that cause an action to be considered challenging. The study of the influence of these psychological processes will allow us to better understand, though possibly not to justify, some decisions with apparent dubious moral consequences.

Finally, the empirical support of the awe-quixoteism hypothesis may also have promising applied value. First, eliciting quixoteism while designing a situation that presents a coherent means-end relationship may promote the performance of relatively exceptional prosocial behaviors such as political activism and social entrepreneurship (Thomas & McGarty, 2018; Vecchione et al., 2015). Indeed, in a recently published choral paper regarding how social and behavioral science can be used to support an effective response to the COVID-19 pandemic (Van Bavel et al., 2020), the authors point out that “some research has revealed that people strongly prioritize local interests over global (or international) interests”. Among other strategies to overcome this obstacle, they propose “guiding individuals towards the mindsets that this illness is manageable, their bodies are capable, and that this can be an opportunity to make positive changes in the world”; we advocate adding the awe-quixoteism link to the repertoire of the processes that may promote that kind of mindset that is effective for dealing with worldwide difficulties.

## Data Availability

The data that support the findings of this work are openly accessible in Open Science Framework at http://doi.org/10.17605/OSF.IO/8AD5F.

## Footnotes

1 The Social Progress Index (SPI) measures the extent to which countries provide for the social and environmental needs of their citizens. It includes fifty-four indicators in the areas of basic human needs, foundations of well-being, and opportunity to progress.

2 We reasoned that measuring the emotional response right before responding to the dilemma would have disrupted the natural stream of the events, dampening the priming effect and introducing experimental demands. Specifically, asking to answer an emotional scale after the emotional prime and before the measure of the dependent variable could, first, affect to the fluent influence of the elicited emotions and, second, lead the participants to think about what hypotheses were being tested. In sum, they could answer to the dilemma more in line with the reported emotions and the hypotheses guessing than with the actual feelings.

3 All the data presented in this work were collected before the COVID-19 pandemic.
